# Novel lichen-dominated hypolithic communities in the Namib Desert

**DOI:** 10.1007/s00248-021-01812-w

**Published:** 2021-07-27

**Authors:** Asunción de los Ríos, Isaac Garrido-Benavent, Alicia Limón, Errol D. Cason, Gillian Maggs-Kölling, Don Cowan, Angel Valverde

**Affiliations:** 1 Biogeochemistry and Microbial Ecology Department, Museo Nacional de Ciencias Naturales, CSIC, Serrano 115 dpdo, 28006 Madrid, Spain; 2 Departament de Botànica i Geologia, Facultat de Ciències Biològiques, Universitat de València (UV), C. Doctor Moliner 50, 46100 Burjassot, València Spain; 3 Department of Animal Science, University of the Free State, Bloemfontein, South Africa; 4 Gobabeb-Namib Research Institute, Walvis Bay, Namibia; 5 Centre for Microbial Ecology and Genomics, Department of Biochemistry, Genetics and Microbiology, University of Pretoria, Pretoria, 0002 South Africa; 6 Instituto de Recursos Naturales y Agrobiología de Salamanca (IRNASA-CSIC), C/ Cordel de Merinas 40-52, 37008 Salamanca, Spain

**Keywords:** Cyanobacteria, Lichens, Lithobionts, Habitat specificity, Dew, Fog, *Stellarangia*, *Buellia*

## Abstract

The ventral surfaces of translucent rocks from hot desert pavements often harbor hypolithic microbial communities, which are mostly dominated by cyanobacteria. The Namib Desert fog belt supports extensive hypolithic colonization of quartz rocks, which are also colonized by lichens on their dorsal surfaces. Here, we aim to evaluate whether lichens colonize the ventral surface of the rocks (i.e., show hypolithic lifestyle) and compare the bacterial composition of these coastal hypolithic communities with those found inland. Fungal DNA barcoding and fungal and bacterial Illumina metabarcoding were combined with electron microscopy to characterize the composition and spatial structure of hypolithic communities from two (coastal and inland) areas in the Namib Desert. We report, for the first time, the structure and composition of lichen-dominated hypolithic communities found in the coastal zone of the Namib Desert with extensive epilithic lichen cover. Lichen modified areoles with inverted morphology of the genus *Stellarangia* (three lineages) and *Buellia* (two lineages) were the main components of these hypolithic communities. Some of these lineages were also found in epilithic habitats. These lichen-dominated hypolithic communities differed in structural organization and bacterial community composition from those found in inland areas. The hypolithic lichen colonization characterized here seems not to be an extension of epilithic or biological soil crust lichen growths but the result of specific sublithic microenvironmental conditions. Moisture derived from fog and dew could be the main driver of this unique colonization.

**Supplementary Information:**

The online version contains supplementary material available at 10.1007/s00248-021-01812-w.

## Introduction

Life in desert soil is principally constrained by water availability. Open desert soils are relatively depauperate habitats that support low-biomass microbial communities [1]. In contrast, the ventral surfaces of translucent rocks (mainly quartz or marble) often harbor hypolithic communities that constitute substantial standing biomass [2–4]. Indeed, hypolithic microbial communities are considered hotspots of primary productivity and organic matter accumulation in hyper-arid deserts [5]. Under these translucent rocks, microbial communities exist in microrefugia with less stringent environmental conditions than open soils, where the overlying lithic substrate provides protection against high incident UV fluxes, generates thermal buffering, and enhances moisture availability [6].

The most common hypolithic communities in hot deserts are those dominated by cyanobacteria [2, 4, 6, 7]. Moss-dominated hypolithic communities have also been reported in the Mojave Desert [8] and in the cold Antarctic Dry Valleys [3, 9, 10]. In contrast, fungal-dominated hypolith communities have only been reported in the Antarctic Dry Valleys [9], although different fungal taxa have been detected in cyanobacteria and moss-dominated lithobiontic communities from different deserts [11–13]. These types of hypoliths may reflect different successional stages [9].

Lichenized fungi are conspicuous in both hot and cold deserts as components of soil crusts [14] or saxicolous (epilithic) communities [15–17]. As poikilohydric organisms, lichens show physiological and anatomical adaptations that allow them to survive on desert soils and rock surfaces, such as thick cortical layers, the production of photoprotective pigments, LEA-like proteins, and the development of potent antioxidant mechanisms [18]. Lichens have also frequently been reported to colonize hidden and protected endolithic microhabitats in desert ecosystems [16, 17, 19]. In contrast, the presence of lichens in hypolithic habitats has, until now, been underexplored. Previously, the colonization of the ventral surfaces of quartz rocks by the cyanolichen *Peltula inversa* has been described in the Namib Desert [20], and chlorolichens (i.e., lichens with eukaryotic green microalgae as photobionts) have also been found underneath small translucent flints in dew-dominated desert areas such as the highlands of Central Asia [21].

In coastal deserts, the occurrence of fog and dew, together with the existence of stable gravel plains, facilitates the development of lichens in areas where rainfall is insufficient to support extensive vascular plant growth [22, 23]. Indeed, the Namib Desert fog belt supports the world’s most extensive lichen cover and a very high diversity of lichens [24]. This extensive lichen colonization gives rise to the unique and renowned “lichen fields” [25], defined by Jürgens and Niebel-Lohmann [26] as “plant formations of considerable surface area, in which epilithic to epipsammic (terricolous) lichens play the dominant role with respect to structure, cover and biomass, if compared with ferns and seed plants.” Quartz rocks embedded in coastal desert pavements in the Namib Desert are frequently colonized by epilithic lichens [27].

In considering whether the communities present on the dorsal surfaces of coastal quartz rocks extended to the ventral surfaces, we investigated hypolithic microbial (fungal and bacterial) colonization using a combination of electron microscopy and rRNA gene sequencing. We also compared the hypolithic microbial communities of these coastal (fog-dominated) rocks with those found in inland (rainfall-dominated) zones to evaluate their uniqueness. Water relations in the inland areas in the Namib Desert are characterized by occasional rainfall events rather than frequent fog events [28], and their hypolithic microbial communities are dominated by cyanobacteria [4, 29]. We hypothesized that translucent rocks in these two areas, subjected to contrasting abiotic conditions and biotic structures, would support different hypolithic communities in terms of composition and predicted functions.

## Materials and methods

### Sample collection

Twenty-five quartz rocks showing visible hypolithic growth were collected on 15–16 April 2015 from an area (S23° 3′ 23″, E14° 38′ 42″) near the coast of the Namib Desert (105 m asl). This area corresponded to lichen field I [25], which is characterized by a dominance of crustose lichens on quartz gravel with a lichen density below 20% and by frequent fog events (Supplementary Fig. 1). Rocks (in the size range of 4–6-cm wide, 4–5-cm long, and 1–2-cm high) were randomly collected, at a minimum distance of 1 m from each other, within a 5-m radius site and stored in sterile Whirl–Pak bags. Ten rocks were used for targeted morphological and molecular identification of lichenized fungi, five for electron microscopy analysis, and ten rocks for community structure comparison with ten rocks found inland by high-throughput sequencing. Five additional rock samples exhibiting hypolithic growth from the inland area (S23°33′32″, E15° 02′15″), near the Gobabeb Research and Training Station (409 m asl), were collected for comparative microscopy studies (Supplementary Fig. 1). In total, 40 rocks were collected (25 near the coast and 15 inland).

### Identification of lichenized fungi

Preliminary selection and identification of lichenized fungi was done visually based on the seminal work by [24] on the lichen flora of the Namib Desert. Confirmation of the identity of taxa at the genus level was done microscopically using hand-cut sections of ascomata mounted in water that were observed using a Zeiss Axioplan 2 microscope fitted with ‘‘Nomarski’’ differential interference contrast (DIC). This was especially important for determining the identity of crustose species belonging to the complex genus *Buellia* De Not. Specimens were deposited in the Royal Botanical Garden of Madrid (MA).

DNA isolation from these selected epilithic and hypolithic lichen growths was performed as follows. Lichen areoles (*Buellia* spp. and *Stellarangia* spp.), lobes (*Xanthoparmelia* spp.), or hypolithic growths (species not identifiable morphologically) were first ground in liquid nitrogen with a mortar and pestle. Total DNA was then extracted using the Speedtools Tissue DNA kit (Biotools® B&M Labs., S.A), following the manufacturer’s recommendations. The fungal DNA barcode nuclear ribosomal Internal Transcribed Spacer, which includes the subregions ITS1, 5.8S, and ITS2, was amplified from each sample. Three markers were also sequenced for *Stellarangia* spp. samples: the nuclear large subunit ribosomal RNA (LSU), the mitochondrial small subunit ribosomal RNA (mtSSU), and the RNA polymerase II largest subunit (*RPB1*). The primers used are shown in Supplementary Table 1. PCR amplifications were carried out using the Illustra Ready-To-Go GenomiPhi V3 DNA amplification kit (GE Healthcare Bio-Sciences, Pittsburgh, Pennsylvania, USA) following the manufacturer’s instructions. PCR conditions for ITS amplifications were as follows: an initial 4 min heating step at 94 °C, followed by 30 cycles of 1.15 min at 94 °C, 1.30 min at 52 °C, and 1.45 min at 72 °C, followed by a final extension step of 10 min at 72 °C, after which the samples were kept at 4 °C. Negative controls lacking DNA were run to check for contamination. Amplicons were purified and cleaned using the QIAGEN quick spin columns (Qiagen**®**). Both complementary DNA strands were sequenced at MACROGEN. Raw electropherograms were manually checked, trimmed, and assembled using SeqmanII v.5.07^©^ (Dnastar Inc.). GenBank accession numbers are in the Supplementary information (Table S1).

Species identification was first approximated by comparing the ITS sequences with nucleotide data deposited in the GenBank database (http://www.ncbi.nlm.nih.gov/) using the BLAST online tool. Secondly, highly similar ITS sequences of *Buellia* spp. and *Stellarangia* spp. were retrieved and aligned independently with the software MAFFT v.7.308 [30]. Ambiguously aligned regions in the *Buellia* spp. dataset were automatically removed using the least stringent parameter options with GBlocks v.0.91b [31]. In *Stellarangia* spp., alignments of LSU, mtSSU and *RPB1* sequences were identically built and concatenated to the ITS sequence dataset. Phylogenetic analyses were conducted under a maximum likelihood (ML) and Bayesian inference (BI) scenarios. The online version of RAxML-HPC2 hosted at the CIPRES Science Gateway [32, 33] was chosen to infer ML phylogenies, and 1000 bootstrap pseudoreplicates were calculated to evaluate nodal support. The MrBayes analyses were conducted with two parallel, simultaneous four-chain runs executed over 1 × 10^8^ generations starting with a random tree, and sampling after every 1 × 10^4^ steps. The first 25% of data were discarded as burn-in, and the 50% majority-rule consensus tree and corresponding posterior probabilities were calculated from the remaining trees. Optimal substitution models for the two partitions within the nrITS (ITS1 + 2, 5.8S; *Buellia* spp. dataset) and five partitions (ITS1 + 2, 5.8S, LSU, mtSSU, *RPB1*; *Stellarangia* spp. dataset) used in the above analyses (Supplementary Table 2) were inferred with PartitionFinder v.1.1.1 considering a model with linked branch lengths and the Bayesian information criterion (BIC). Average standard deviation of split frequencies (ASDSF) values below 0.005 and potential scale reduction factor (PSRF) values approaching 1.00 were considered indicators of chain convergence in the Bayesian analyses. As for tree nodal support, nodes showing Bootstrap support (BS) values equal or higher than 70% (RAxML analyses) and Bayesian posterior probabilities (PP) equal or higher than 0.95 (MrBayes analyses) were regarded as significantly supported. Phylogenetic trees were visualized in FigTree v.1.4 (available at http://tree.bio.ed.ac.uk/software/tracer/), and Adobe Illustrator CS5 was used for artwork.

### High-throughput sequencing of hypolithic communities

Genomic DNA was extracted from hypolithic microbial biomass scraped from quartz rocks using the PowerSoil® DNA Isolation Kit (MO BIO laboratories, Carlsbad, CA, United States). DNA quality and concentration were measured using a NanoDrop ND 1000 spectrophotometer (Thermo Fisher Scientific™). For bacterial DNA amplification, we followed the bacterial 16S rRNA Illumina Amplicon Protocol recommended by the Earth Microbiome Project (available at http://www.earthmicrobiome.org/protocols-and-standards/16s/), using the primer pair 515F (5′-GTGYCAGCMGCCGCGGTAA-3′) and 806R (5′-GGACTACNVGGGTWTCTAAT-3′), which amplifies the V4 region of the 16S ribosomal RNA gene [34]. Fungal library preparation was performed with a two-step PCR method, using the ITS1F-KYO1 and ITS2-KYO2 primer set that spans the ITS1 region. To this end, PCR amplifications for each sample were conducted in triplicate, quantified using Quant-iT™ PicoGreen® dsDNA Assay Kit (Invitrogen) and pooled at equimolar amounts. A no-template sample was included during library preparation as a control for extraneous nucleic acid contamination. Amplicon products containing sample-specific barcodes were quantified using the Illumina library Quantification Kit ABI Prism® (Kapa Biosystems), pooled together in equal concentrations (240 ng of DNA per sample), and then cleaned using the QIA quick PCR purification kit (QIAGEN). Subsequently, the DNA pool was diluted to a final concentration of 4 nM and then denatured and diluted to a final concentration of 4 pM in 15% PhiX. Finally, the DNA library was loaded in MiSeq Illumina and run using the version 2 module, 2 × 250 pair-end, following the manufacturer’s instructions. Raw reads were demultiplexed, and barcode sequences were removed by the sequencing centre. Both amplicon libraries were generated at the ASU Genomics Core (Arizona State University, USA).

### Amplicon quality processing, clustering, and classification

High-throughput sequence analysis and bioinformatic processing was performed according to [35]. Briefly, 16S rRNA and ITS sequence datasets were pre-processed and trimmed using PrinSeq-lite v0.20.4 to obtain an average quality score ≥ 25 using a 7 nt window with a 4 nt step. All sequences shorter than 100 bp were filtered out. Paired end reads were merged using PEAR 0.9.6 [36]. Quality filtered reads were analyzed using QIIME v1.9.1 [34]. Chimeric sequences were identified and removed using Usearch v6.1.544 against the RDP “Gold” database [37] for bacteria/archaea and the UNITE database for fungi [38]. Operational taxonomic unit (OTU) picking were carried out at 99% sequence identity against the SILVA 132 database [39] for the bacterial/archaeal 16S rRNA and the UNITE v 8.2 [38] for the fungal ITS data.

The newly obtained raw high-throughput sequencing data, for both fungi and bacteria, was deposited into the NCBI Sequence Read Archive (SRA) database (BioProject ID: PRJNA734915).

### Comparing coastal and inland hypolithic bacterial communities

To compare the bacterial communities from coastal hypoliths with those from inland sites, we used 16S rRNA gene high-throughput sequencing data obtained above and from a previous study [40]. For OTU comparisons, given that the sequencing data from [40] was obtained with primers 27F/519R on a Roche 454 FLX titanium instrument, we used the OTUs closed-reference picking protocol implemented in QIIME v1.9.0 [34]. The OTUs closed-reference protocol allows comparison of sequences obtained using primers that targeted different regions of the 16S rRNA gene and different sequence platforms [41]. Functional prediction based on OTU representative sequences was performed using PICRUSt2 version 2.1.4_b [42].

### Statistical analyses

Differences in alpha diversity (Inverse Simpson, Shannon and observed number of OTUs) and relative abundance (at phylum and family levels) between coastal and inland bacterial hypolithic communities were assessed using Kruskal–Wallis tests. Differences in bacterial community composition and predicted functions between coastal and non-coastal hypolithic communities were based on Bray–Curtis dissimilarities using permutational multivariate ANOVA (PERMANOVA) [43], implemented using the *adonis* function in the R package vegan [44]. Within-group variance in community composition and predicted function was performed using the *betadisper* function in vegan. Changes in gene abundance between coastal and non-coastal hypolithic bacterial communities were tested using Welch’s t-tests implemented in STAMP [45].

### Characterization of hypolithic communities by scanning electron microscopy with backscattered electron imaging (SEM-BSE)

The rock-microorganism interface was observed by scanning electron microscopy with backscattered electron imaging (SEM-BSE) [46]. Briefly, rock fragments containing hypolithic communities were fixed in glutaraldehyde (3% v/v) and osmium tetroxide solutions (1% w/v), dehydrated in a graded ethanol series (from 30 to 100% v/v), and embedded in LR-White resin. The resulting blocks were finely polished, carbon coated, and observed using a FEI INSPECT 105 SEM microscope. Microprobe analyses was performed using an Oxford Instruments INCA X-act Energy Dispersive Spectrometer (EDS) microanalytical system during SEM observations.

## Results

### Visual identification of epilithic and hypolithic colonizers

**Fig. 1:**
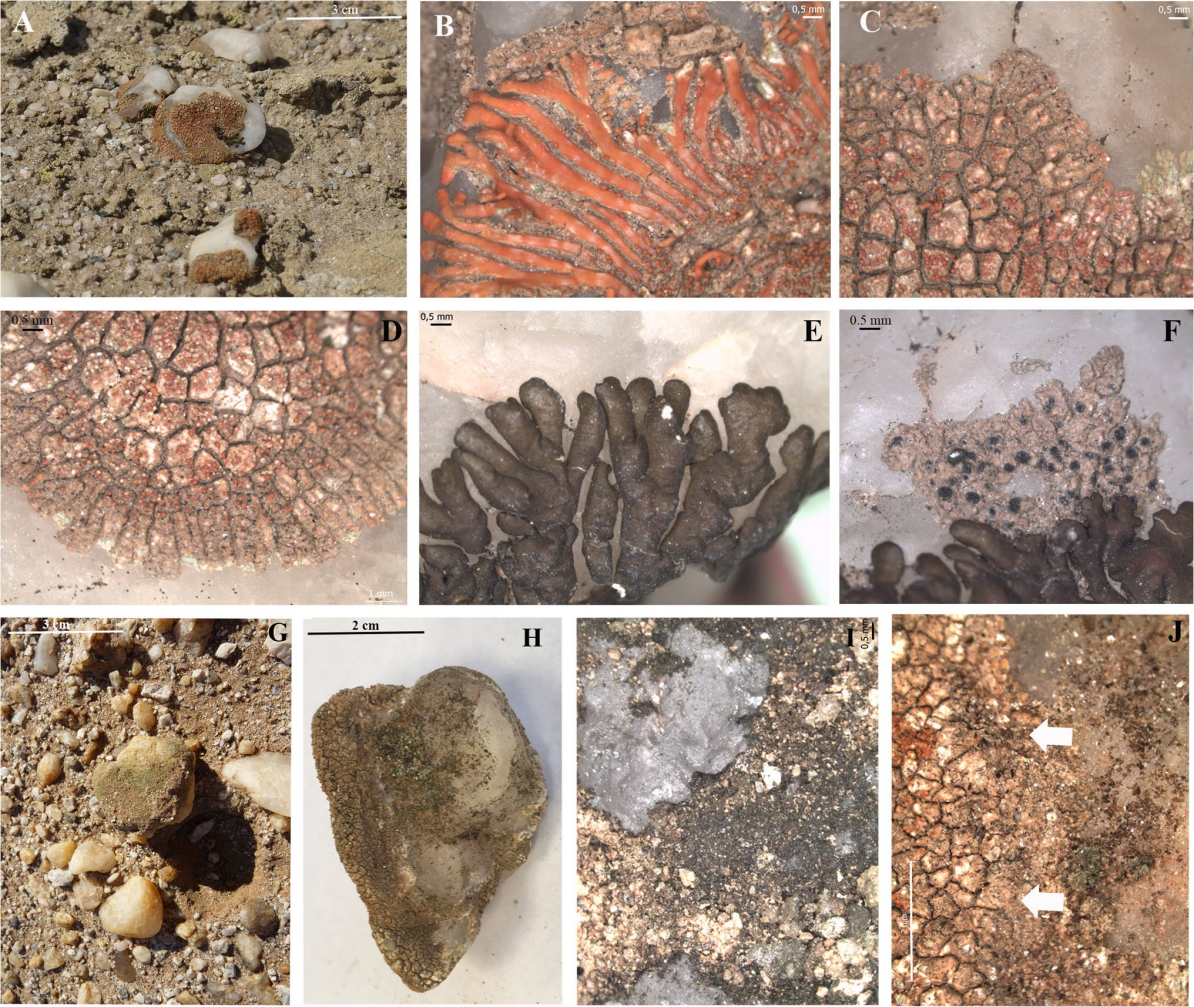
**A** Quartz pavement in the coastal area of the Namib Desert supporting epilithic lichen colonization; **B** epilithic *Stellarangia namibensis*; **C** epilithic *S. testudinea*; **D** epilithic *Stellarangia* sp.; **E** epilithic *Xanthoparmelia* sp.; **F** epilithic *Buellia* sp.; **G** quartz rock supporting green hypolithic community from the inland area; **H** quartz rock supporting green hypolithic community from the coastal area; **I** black hypolithic growth from the inland area; **J** hypolithic *Stellarangia* sp. areoles (arrows) from the coastal area

The upper surface of most quartz rocks from the coastal area was colonized by lichens (Fig. 1A). *Stellarangia* spp. (Fig. 1B-D) were the lichenized fungi more commonly found at epilithic locations, but species in *Xanthoparmelia* (Fig. 1E) and *Buellia* (Fig. 1F) were also observed. In contrast, the dorsal surfaces of quartz rocks from the inland area showed no lichen colonization. Hypolithic colonization was detected in quartz rocks from both localities, but two different types of communities were distinguished, cyanobacteria-dominated communities and lichen-dominated communities. Greenish (Fig. 1G–H) and black (Fig. 1I) hypolithic communities dominated by cyanobacteria were found in both, coastal and inland samples. Hypolithic communities harboring modified lichen areoles (arrows in Fig. 1J) were found only in the coastal area.

### Targeted molecular and phylogenetic identification of epilithic and hypolithic fungal colonizers

**Fig. 2:**
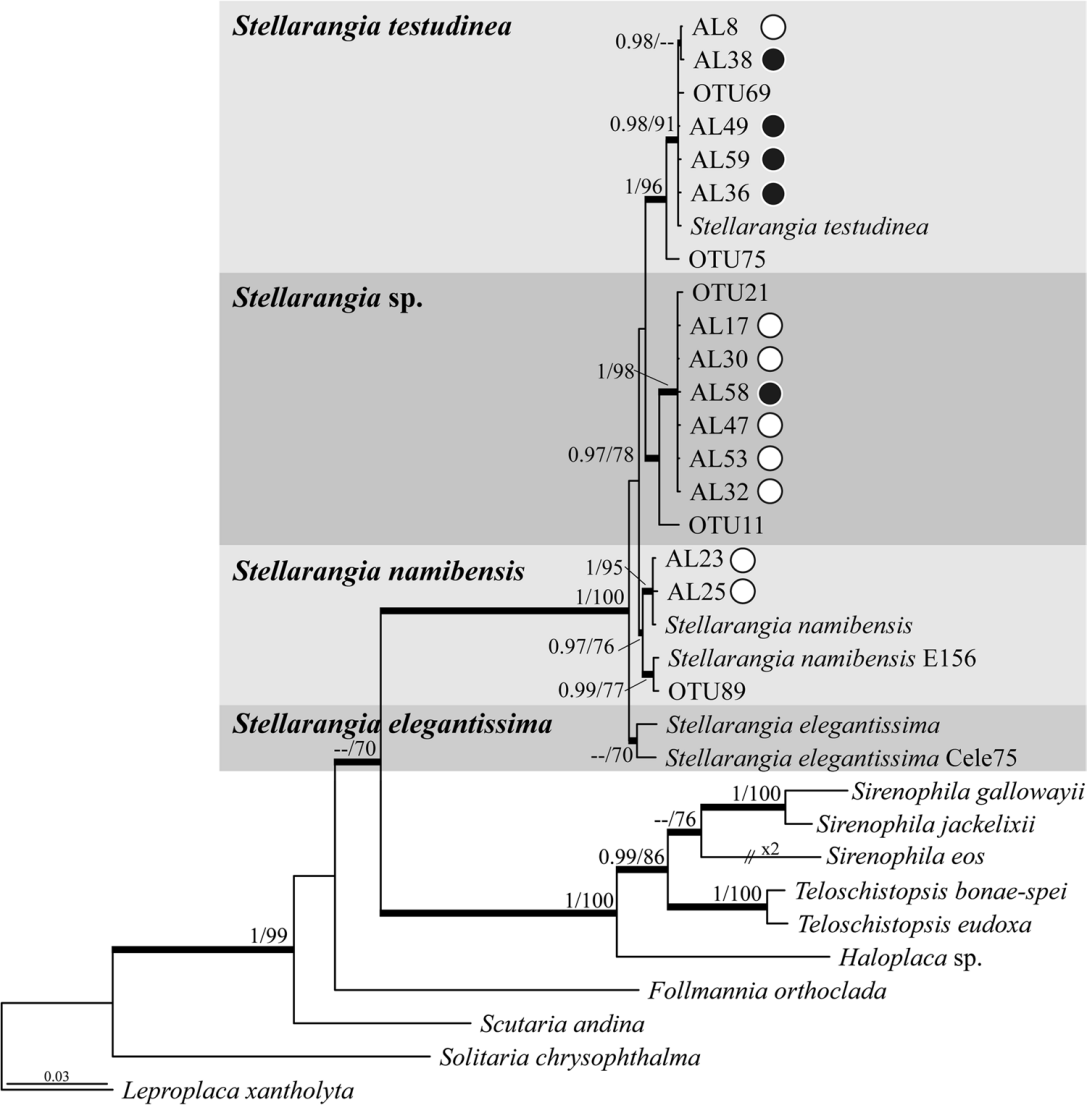
Phylogram depicting the evolutionary relationships of species within the lichenized fungal genus *Stellarangia* based on a four loci (ITS, LSU, mtSSU, and *RPB1*) dataset obtained using Sanger sequencing. The represented topology was obtained under a Bayesian framework using MrBayes. Sequences generated from lichen thalli are labelled with “AL,” whereas those obtained in the high-throughput study (ITS data) correspond with the OTUs. Data from the remaining species depicted in the tree was obtained from GenBank (see Supplementary Table 4). Posterior probabilities (PP, Bayesian analyses) and bootstrap support (BS, RAxML analyses) are represented on branches leading to nodes. Black dots denote hypolithic habitats and white dots epilithic habitats

**Fig. 3:**
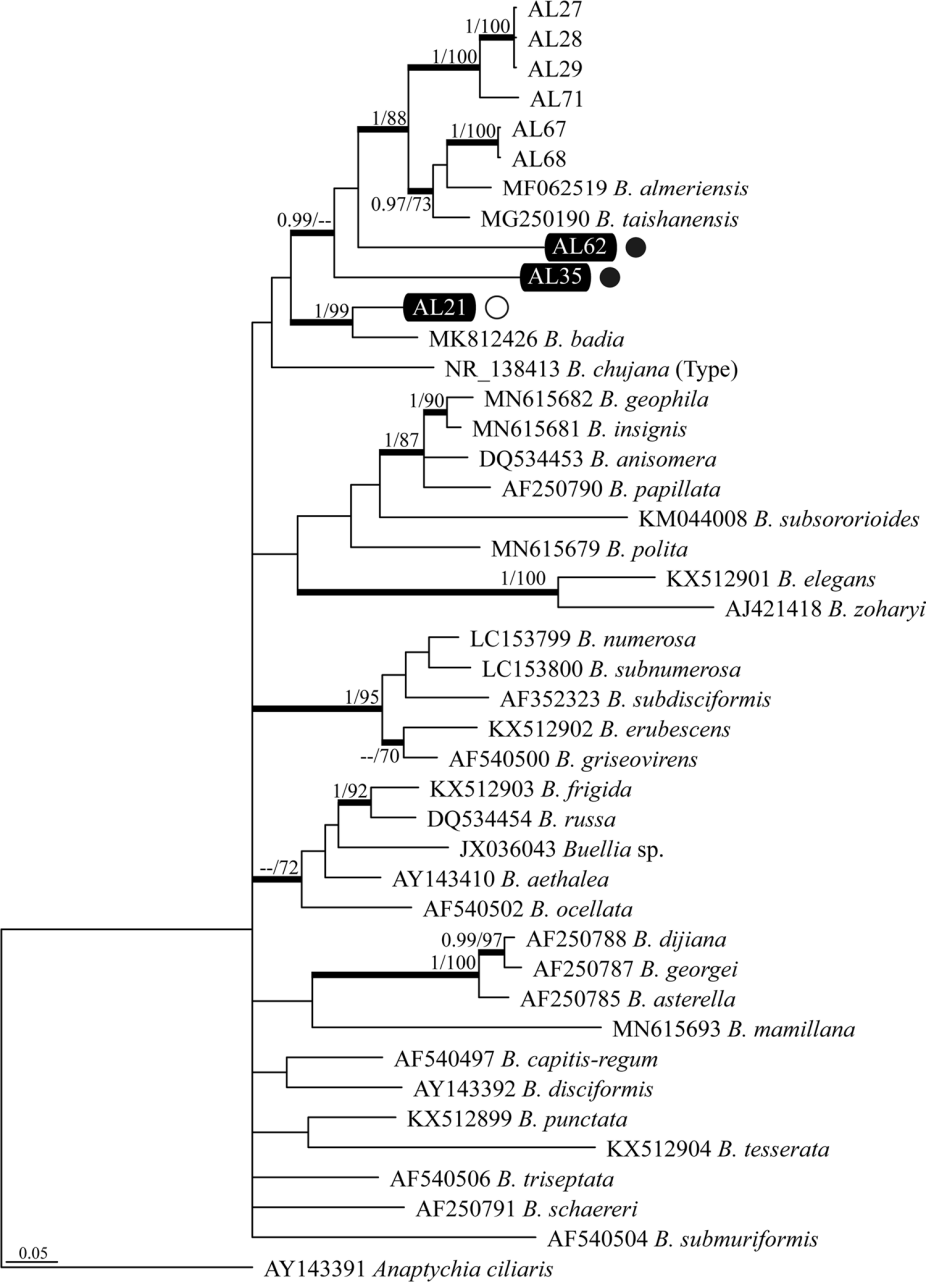
Phylogram depicting the evolutionary relationships of species within the lichenized fungal genus *Buellia* based on Sanger sequencing of the ITS. The represented topology was obtained in the Bayesian analysis. Sequences generated from lichen thalli are labelled with “AL”; the ones within a black rectangle were obtained from epilithic thalli. Data from the remaining species depicted in the tree was obtained from GenBank (see Supplementary Table 5). Posterior probabilities (PP, Bayesian analyses) and bootstrap support (BS, RAxML analyses) are represented on branches leading to nodes. Black dots denote hypolithic habitats and white dots epilithic habitats

Three distinct genetic lineages of *Stellarangia* spp. were revealed by BLAST searches of the ITS sequences obtained from epilithic lichen areoles. One such lineage represented the species *Stellarangia namibensis* (Kärnefelt) Frödén, Arup & Søchting and another *S. testudinea* (V. Wirth & Kärnefelt) Frödén, Arup & Søchting (Supplementary Table 3)*.* Our ITS sequences clustered into statistically well-supported clades representing these two taxa in our four-loci ML and Bayesian phylogenetic reconstructions (Fig. 2). The third *Stellarangia* lineage formed an independent and well-supported (PP = 1; BS = 98%) clade in our phylogenies. However, relationships of this lineage with clades representing the other *Stellarangia* species were not supported (Fig. 2). The ITS sequence generated from an epilithic thallus of a *Buellia* species (sample AL21) showed the closest match to *B. badia* (Fr.) A. Massal with a sequence similarity of 91% (Supplementary Table 3), whereas samples AL35 and AL62 were only distantly related to any other known *Buellia* species. In fact, the former two lineages formed a statistically well-supported clade (PP = 1; BS = 99%) in our ML and Bayesian phylogenetic trees (Fig. 3). Finally, the ITS sequences generated from *Xanthoparmelia* thalli had closest matches to *X. taractica* (Kremp.) Hale, although with a relatively low sequence similarity (91.7%; Supplementary Table 3).

Hypolithic lichen thalli contained species of the genus *Stellarangia* (three distinct lineages) and *Buellia* (two lineages). Hypolithic *Stellarangia* sequences were identical or closely matched to sequences obtained from epilithic *S. testudinea* and *Stellarangia* sp. (Fig. 2). In contrast, ITS sequences of hypolithic *Buellia* spp. were substantially dissimilar to those obtained from epilithic thalli (Supplementary Table 3) and thus were located into an unrelated phylogenetic clade, which was the sister (PP = 1; BS = 88%) to a subclade containing *B. almeriensis* Llimona and *B. taishanensis* Q.D. Wang & Z.F. Jia (Fig. 3)*.*

### High-throughput sequencing of fungal ITS regions from lichen-dominated hypolithic communities

**Fig. 4:**
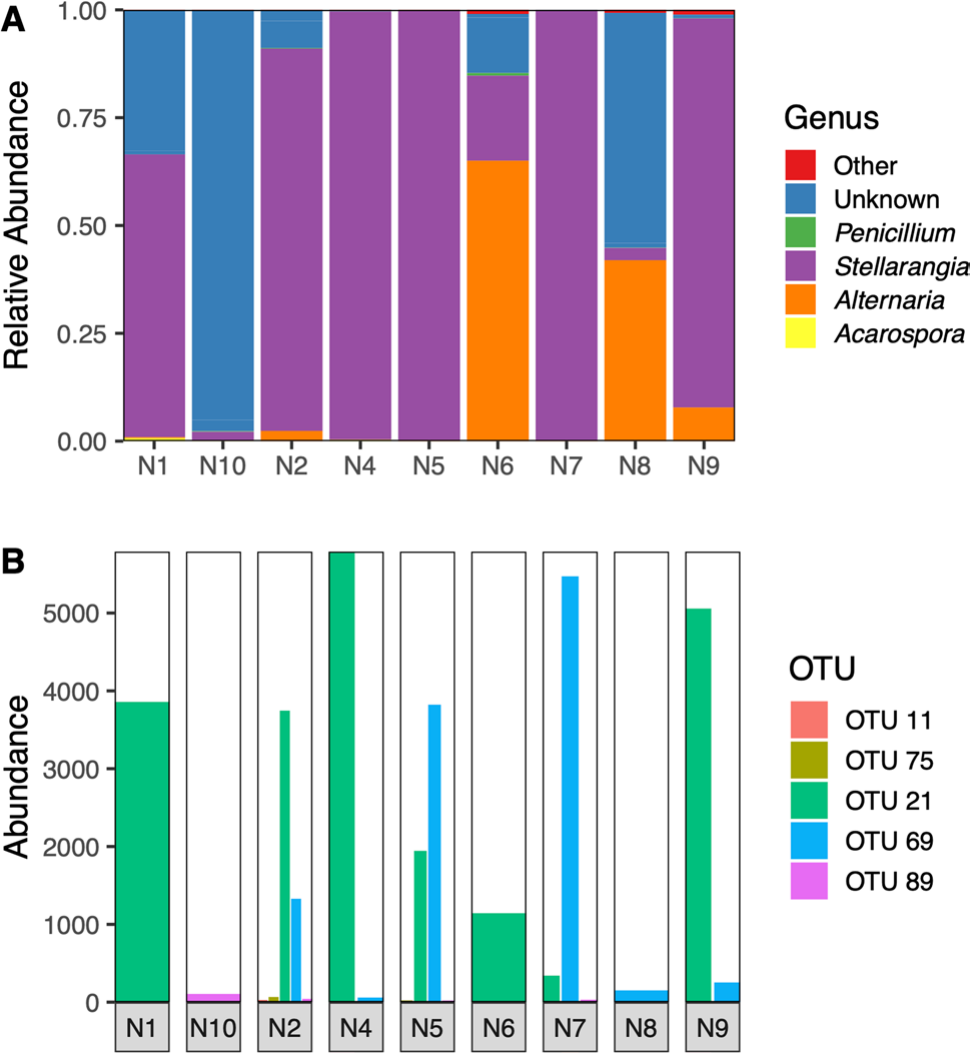
**A** Relative abundance of fungal genera (**A**) and fungal OTUs (**B**) in hypolithic growths from coastal area (N1–N10) obtained through high-throughput Illumina sequencing. N1, N2, N4, N5, and N6 did not show visible epilithic growth, but epilithic *Stellarangia* thalli were observed in samples N7, N9, and N10 and *Xanthoparmelia* sp. in sample N8

The ITS metabarcoding analysis revealed that lichen-forming fungi were present in all analyzed coastal hypolithic communities, including these from rocks without lichen epilithic colonization. These results also confirmed that these communities were mostly dominated by the lichen-forming genus *Stellarangia* (Fig. 4A–B). Phylogenetic analyses (Fig. 2) revealed that some *Stellarangia* OTUs were closely related to *Stellarangia* sp., while others were closely related to *S. testudinea*. In contrast, samples with conspicuous blackish hypolithic growths and scarce lichen thallus development were dominated by OTUs belonging to the non-lichenized fungal genus *Alternaria*.

### Spatial organization of hypolithic communities

**Fig. 5:**
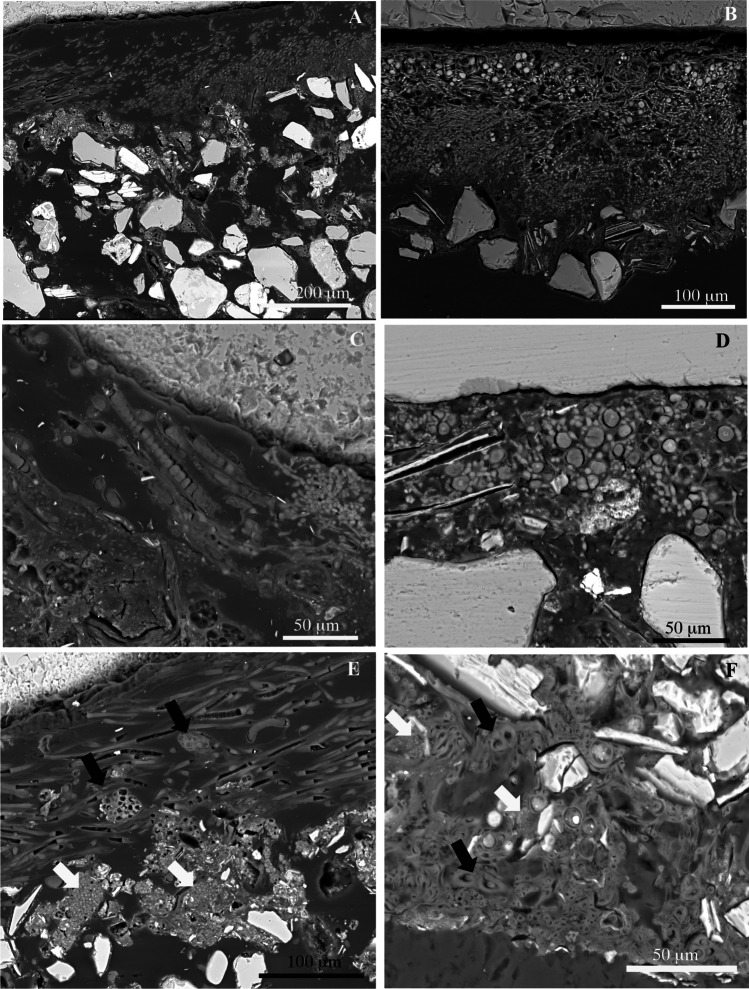
SEM-BSE images of hypolithic growths. **A** Cyanobacteria-dominated hypolithic community from the inland area. **B** Lichen-dominated hypolithic community from the coastal area. **C** Filamentous cyanobacteria closely associated to the ventral surface of quartz rock from the inland area. **D** Algal and fungal symbiont lichen cells in the dense layer of a hypolithic growth from the coastal area. **E** Filamentous cyanobacteria associated to aggregates of putative coccoid cyanobacteria (black arrows) and heterotrophic bacteria (white arrow) in the dense layer of a hypolithic growth from the inland area. **F** Filamentous cyanobacteria (black arrows) and aggregates of coccoid bacteria cells (white arrows) associated to fungal hyphae in the looser layer of hypolithic community from the coastal area

**Fig. 6:**
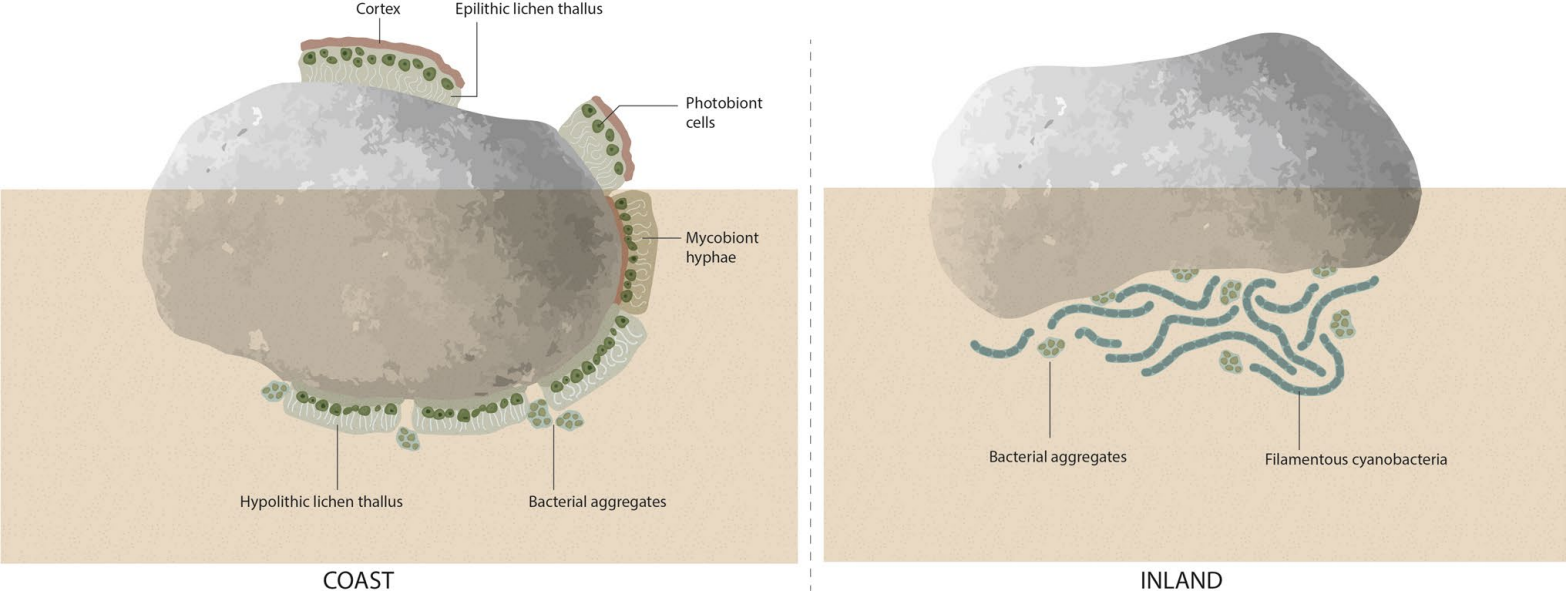
Scheme of lichen-dominated (coastal area) and cyanobacteria (inland area) dominated hypolithic communities

Hypolithic communities from coastal and inland samples shared some spatial structural features (Fig. 5A–B). Dense aggregates with dominance of filamentous cells occupied the proximal layer (with reference to the rock), while a broader loose matrix with more dispersed cells retaining numerous soil mineral fragments occupied the distal layer (Fig. 5A–D). However, hypolithic communities from both areas differed markedly in composition (Fig. 6). In the inland samples, cellular ultrastructure and morphology revealed that filamentous cyanobacteria (Fig. 5C), accompanied by aggregates of putative coccoid cyanobacteria (black arrows in Fig. 5E), dominated the proximal dense layer in hypolithic communities. In contrast, in the coastal samples, lichen symbiont cells were the main components of this dense layer (Fig. 5B). Fungal hyphae appeared associated to green microalgae cells in this dense layer (Fig. 5D), but extended downward (towards the underlying soil) and were associated to different mineral fragments (Fig. 5B). The observed structure resembles a lichen heteromerous thallus organization, with an algal (photobiont) layer differentiated from a medullar layer composed of fungal hyphae. However, this structure lacked an upper cortex. Putative cyanobacteria were frequently observed in the proximity of hyphae from the loose layer at the soil face (black arrows in Fig. 5F). Bacteria-like cell aggregates were detected in both types of hypolithic communities and frequently found in areas dominated by cyanobacteria (white arrows in Fig. 5E–F).

### Anatomical features of hypolithic lichens

**Fig. 7:**
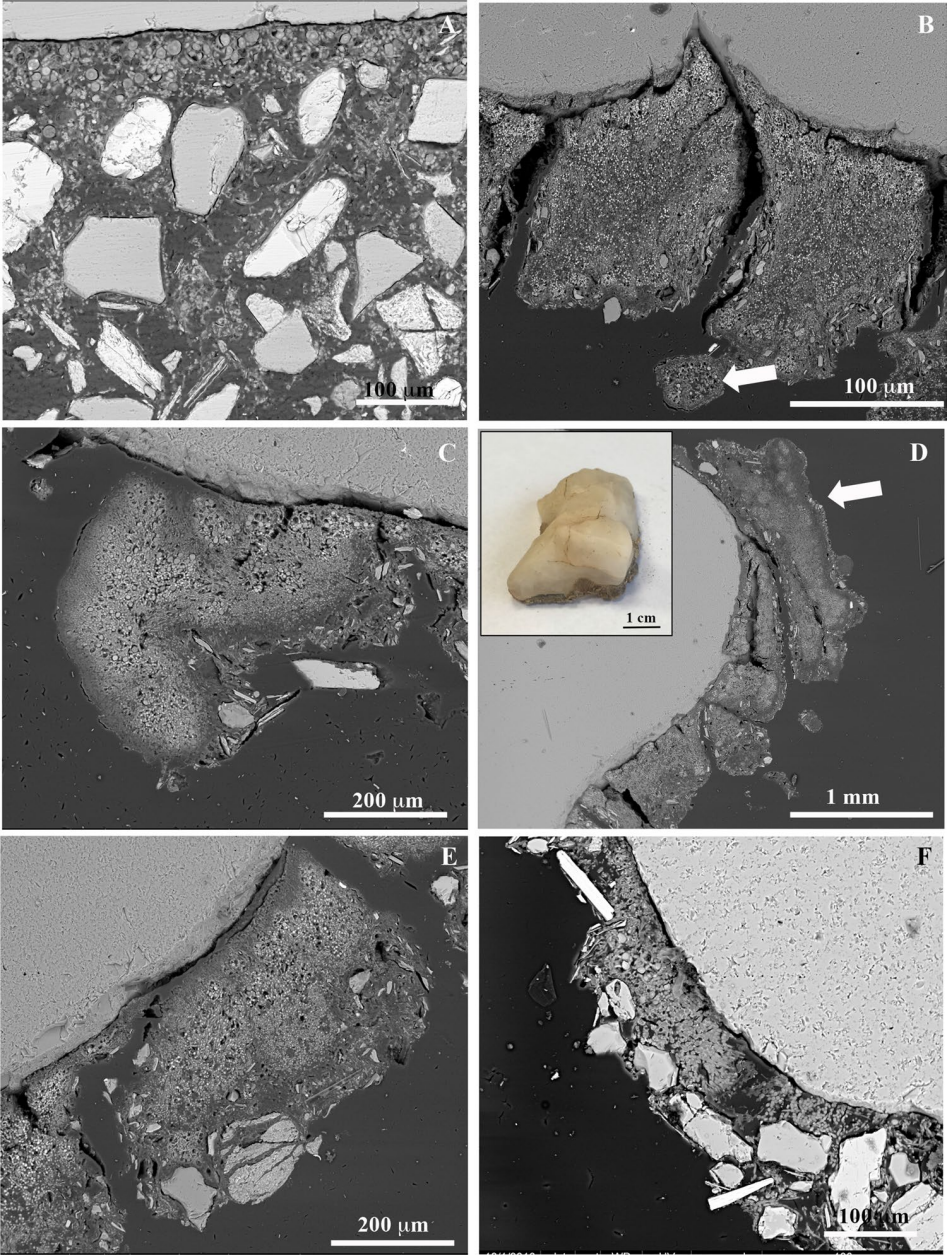
SEM-BSE images of hypolithic lichens. **A** Lichen thalli with inverse internal morphology showing an algal layer exposed toward the quartz rock and lack of upper cortex. **B** *Stellarangia testudinea* areoles showing isidia (arrow) in the lichen surface exposed to the soil. **C** Lichen hypolithic growth showing at the ventral face a section with inverse internal morphology and a growth expansion orientated toward the soil surface. **D** Lichen areoles expanding to the lateral margins of the rocks corresponding to the rock showed in the inset, showing gradual changes in their anatomy. Arrow points to lichen areole at soil surface with upper cortex and non-inverse internal morphology. **E** Lichen areoles at the lateral margin of the rocks with inverse morphology and thin upper cortex. **F**, Associations of algal and fungal lichen symbiont cells without a clear lichen thallus structure

A common feature of lichens found on the ventral surfaces of quartz rocks was an inverse internal morphology, with an algal layer exposed toward the quartz rock surface (Fig. 6, Fig. 7A–B) and no upper cortex (Fig. 7A). In areoles of hypolithic *Stellarangia testudinea*, algal cells were also observed within isidia (i.e., lichen asexual propagule) in the lichen surface exposed to the soil (black arrow in Fig. 7B). Some lichen areoles grew from the hypolithic lichen thallus toward the surface (Fig. 7C). In fact, lichens expanded from the center to the lateral margins of the rock (Fig. 7D) showing gradual changes in their anatomy (Fig. 6). Lichen areoles showed an upper cortex in the lateral margins of rocks, either very thin and orientated toward the rock in areas under the soil surface (Fig. 7E) or broad and orientated opposite to the rock surface above the soil surface (arrow in Fig. 7D). Aggregates of associated algal and fungal cells without a clear lichen thallus structure were also occasionally observed on the margins of the rock surfaces (Fig. 7F).

### Bacterial diversity and composition

**Fig. 8:**
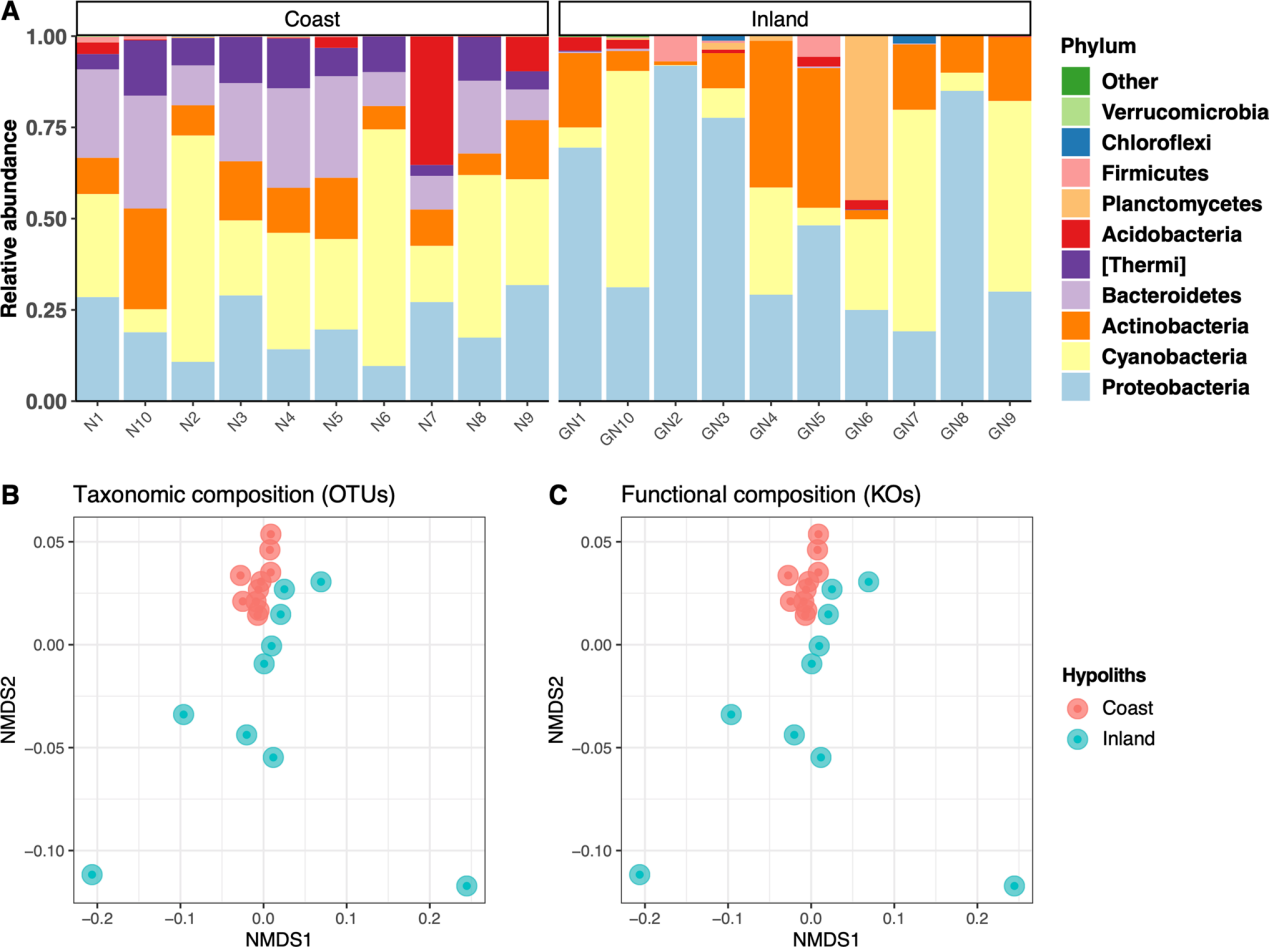
**A** Relative abundance of bacterial phyla in hypolithic growths from coastal (N1–N10) and inland (GN1–GN10) areas obtained through high-throughput Illumina sequencing. Non-metric multidimensional scaling (NMDS) ordination plots (Bray–Curtis dissimilarities) from coastal (N1–N10) and inland (GN1–GN10) sampling points, showing the differences in taxonomic (**B**) and functional (**C**) composition

Bacterial alpha diversity values were similar between the two types of hypoliths (Supplementary Fig. 2). Overall, bacterial communities were dominated by the phyla *Proteobacteria* (36% on average), *Cyanobacteria* (29%), *Actinobacteria* (15%), *Bacteroidetes* (10%), and *Thermi* (5%) (Fig. 8A). The relative abundance of *Proteobacteria* was significantly higher in inland hypoliths compared to coastal hypoliths (Kruskal–Wallis, P < 0.05), while the relative abundance of *Bacteroidetes* and *Thermi* was significantly higher in coastal hypoliths compared to non-coastal hypoliths (Kruskal–Wallis, P < 0.05). At the family level (Supplementary Fig. 3), *Sphingomonadaceae* (9%), *Phormidiaceae* (9%), *Xenococcaceae* (7%), and *Acaryochloridaceae* (5%) mainly contributed to the bacterial community. The relative abundance of the families *Sphingomonadaceae*, *Acaryochloridaceae*, *Geodermatophilaceae*, *Cytophagaceae*, *Bradyrhizobiaceae*, and *Trueperaceae* was significantly higher in coastal hypoliths than in inland hypoliths (Kruskal–Wallis, P < 0.05).

The taxonomic (OTU level) and predicted functional (KO level) community structure of hypolithic bacterial communities from coastal and inland areas differed markedly as revealed by NMDS ordinations (Fig. 8B–C) and PERMANOVA analysis (P < 0.01 in both cases). Furthermore, non-coastal hypoliths were more variable in their taxonomic and functional profiles than coastal hypoliths (permutation dispersion: P < 0.01 in both cases).

## Discussion

Here, we characterize, for the first time, lichen-dominated hypolithic communities from the Namib Desert. Previous research has reported that the distribution of terricolous lichens in the Namib Desert depends on factors such as distance to the coast, elevation, climatic gradients, wind and sand force, substrate, and physiological adaptations [25, 47]. The extensive growth of epilithic and crust forming coastal lichens is thought to be supported by moisture content derived from fog and dew [22, 23, 48]. Water availability has also been considered an important determinant in shaping hypolithic microbial community structure along an inverse fog-rainfall gradient across the central Namib Desert [4]. Hence, the frequent fog and dew events in these coastal areas could also explain the establishment of lichen-dominated hypolithic communities, as the hypolithic habitat increases water retention by shading [2, 20, 49].

Hypolithic habitats in dryland soils provide specific microclimatic conditions that facilitate a level of biocomplexity not possible in surrounding “open” soils [50]. In agreement with this observation, electron microscopy characterization revealed complex spatial structures formed by aggregates of microorganisms from different taxonomic groups trapping soil mineral components and closely associated to the quartz lithic substrate. In fog-dominated coastal areas, filamentous fungi were the main structural components of these communities. Conversely, cyanobacteria were the dominant structural components in communities from inland sites. The structures of lichen-dominated hypolithic communities were more dense than those without lichens, presumably because hypolithic lichen symbionts provide a high degree of thallus organization. In lichen-dominated communities, medullar hyphae were associated to bacterial aggregates and showed clear interactions with soil mineral fragments. A similar relationship was observed between filamentous cyanobacteria and soil mineral fragments in cyanobacteria-dominated communities. Hypolithic aggregates of cyanobacteria and heterotrophic bacteria are generally embedded in a dense matrix of extracellular polymeric substance (EPS), which plays key structural and functional roles [17]. The contribution of EPS to the spatial structures of hypolithic biomass found in the Namib Desert could be more relevant in inland cyanobacteria-dominated communities, because in coastal lichen-dominated communities, bacterial aggregates are restricted to marginal areas at the soil surface. EPS matrices are critically important for the retention of moisture [17], which could be essential for communities in inland (hyper-arid) areas where fog, dew, and rainfall events are very rare and atmospheric relative humidity values are significantly lower than in coastal areas. In contrast to Antarctic moss-dominated hypoliths, in which cyanobacteria frequently associates to the quartz surface between the rock and the moss [10], in the cryptogam-dominated hypolithic community described here, lichen symbionts were closely associated with the quartz rock, and cyanobacteria were not observed at the interface.

The bacterial communities found in coastal and inland hypoliths were clearly different in both taxonomy and predicted function. This is expected, as it has previously been reported that lichens harbor a specific microbiome and that different microbiomes perform different functions [51, 52]. Indeed, *Bacteroidetes*, which showed a higher abundance in lichen-dominated coastal hypoliths compared to cyanobacteria-dominated inland hypoliths, are common in marine and maritime lichens [52]. Bacteroidetes are typically classified as copiotrophs and therefore preferring high nutrient availability [53]. In contrast, *Firmicutes*, *Actinobacteria*, and *Proteobacteria*, which are also ubiquitous in lichens [51, 54], did not display any difference in relative abundance between the two types of hypoliths. Altogether, it seems that both biotic (i.e., the presence of lichens) and abiotic factors (e.g., nutrient levels, water availability) likely determine the composition and predicted functional repertories of the bacterial communities of the two types of hypoliths. The increase in taxonomic and functional similarity in coastal hypoliths is an indication of biotic homogenization, which may indicate a more prominent role of deterministic processes (i.e., habitat filtering) in the assembly of those communities.

The rocks harboring lichen-dominated hypolithic communities were part of a lichen field with high density of saxicolous and crust-forming lichens. However, the hypolithic lichen colonization reported here seems not to be an extension of epilithic or biological soil crust lichen growths. While some rocks showed colonization by the same *Stellarangia* species in both epilithic and hypolithic locations, others only showed hypolithic or epilithic lichen colonization. In addition, connections between hypolithic growths and biological soil crusts as those reported for the cyanolichen *Peltula inversa* [20] were not observed at the studied lichen field. Hence, lichen-dominated hypolithic colonization seems to be the result of specific sublithic microenvironmental conditions. Indeed, hypolithic lichens were adapted to this microenvironment because they showed inverted morphology. The inverted morphology is thought to be an adaptive mechanism to cope with the high irradiance and low water availability that characterize desert soils [20]. Two probably new lichen-forming fungal species were reported in this study, one assigned to the genus *Stellarangia* and the other to the genus *Buellia*, which suggest that these cryptic habitats located under coastal quartz rocks could act as reservoirs of unknown fungal taxa.

## Supplementary Information

Below is the link to the electronic supplementary material.

Supplementary file1 (DOCX 24786 KB)
