# Supplementary material for: Novel lichen-dominated hypolithic communities in the Namib Desert
Source: Microb Ecol. 2021 Jul 27;83(4):1036–48. doi: 10.1007/s00248-021-01812-w (PMC9015988; doi:10.1007/s00248-021-01812-w)
Supplement: Supplementary file 1 — Supplementary file1 (DOCX 24786 KB) [file 248_2021_1812_MOESM1_ESM.docx]

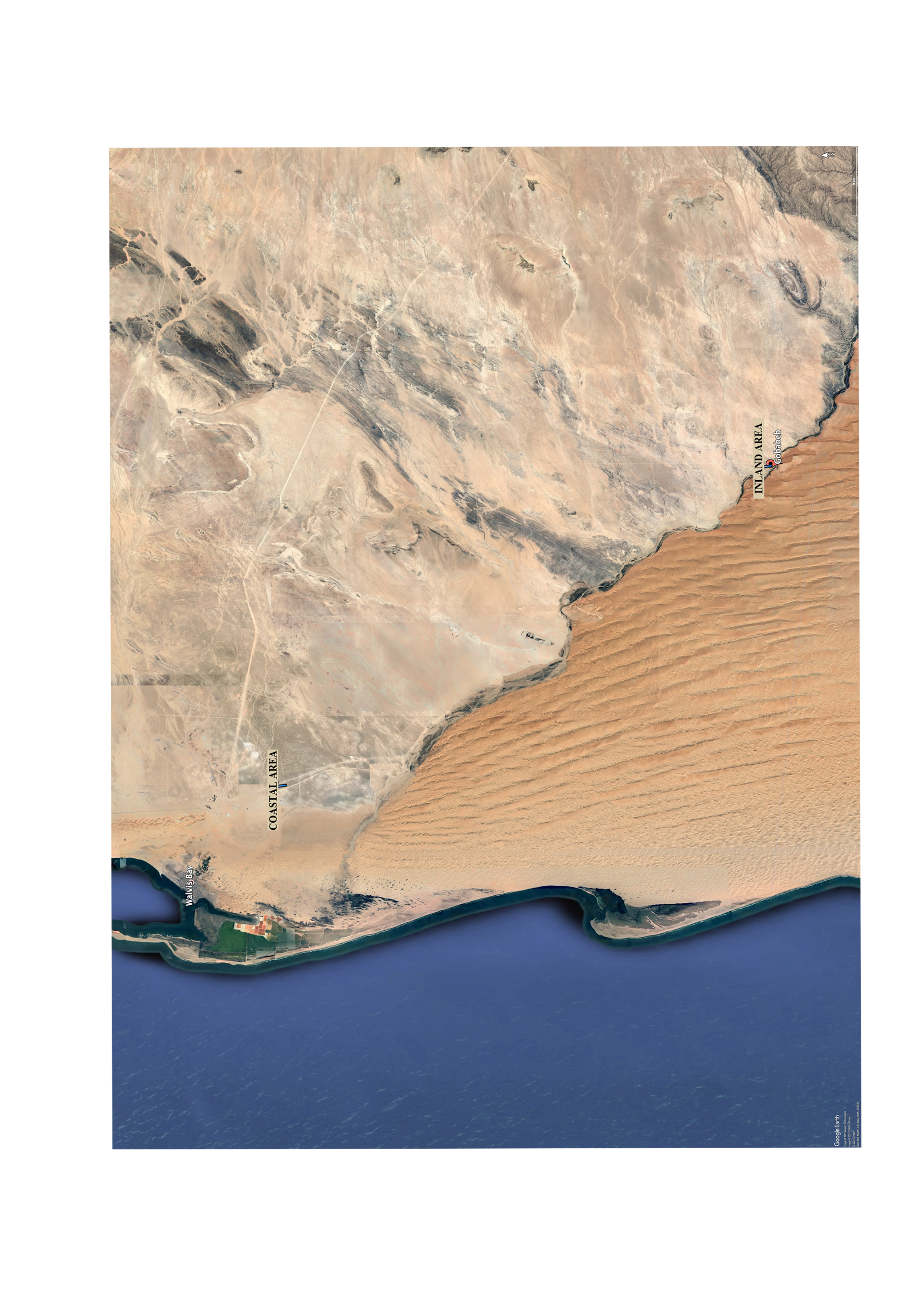


Supplementary Figure 1. Map showing the location of the two sampling areas


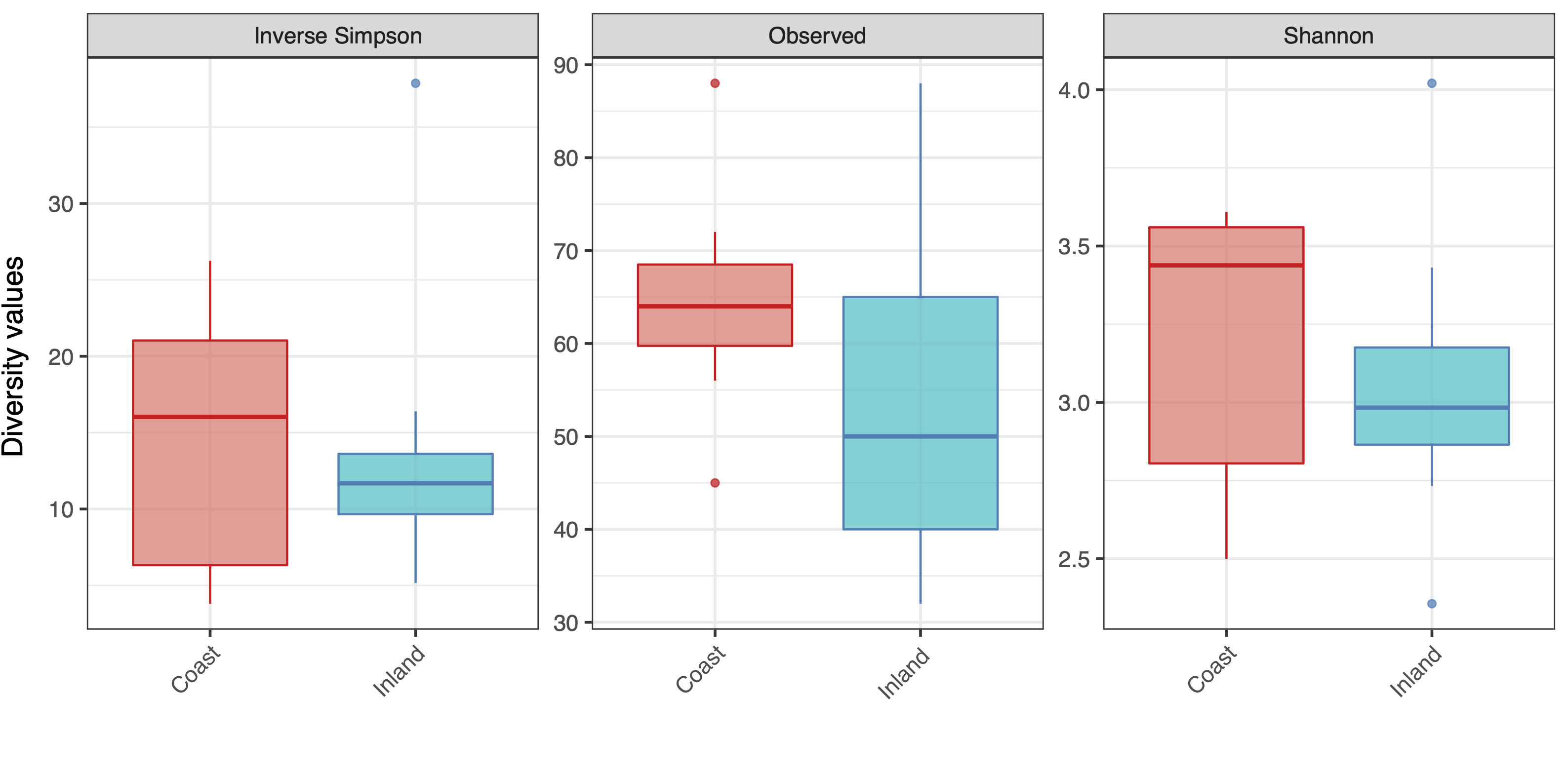


Supplementary Figure 2. Diversity indices of hypolithic (Coast vs Inland) bacterial communities.


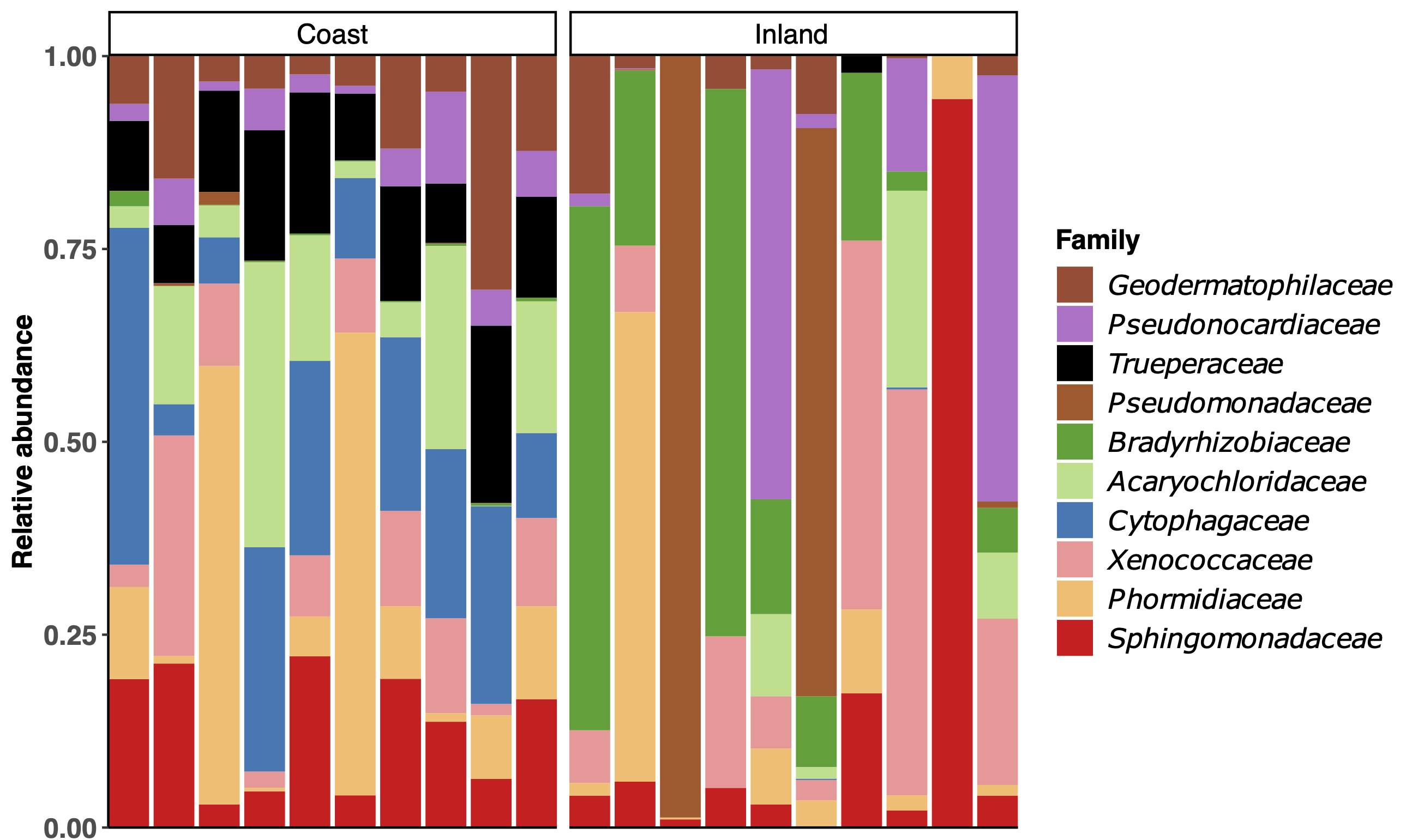


Supplementary Figure3. Relative abundance of bacterial families in hypolithic (Coast vs Inland) communities.

Supplementary Table 1. List of molecular markers sequenced in this study and the primers used for amplification.

| Marker | Primer F (forward) | Primer R (reverse) |
| --- | --- | --- |
| ITS | ITS1F (Gardes and Bruns 1993) | ITS4 (White *et al.* 1990) |
| LSU | LR0R (Rehner and Samuels 1994) | LR5 (Vilgalys and Hester 1990) |
| mtSSU | mrSSU1 (Zoller *et al*. 1999) | mrSSU3R (Zoller *et al*. 1999) |
| *RPB1* | RPB1-Af (Stiller and Hall 1997) | RPB1-Cr (Matheny et al. 2002) |

**Gardes M, Bruns TD**. **1993**. ITS Primers with enhanced specifity for Basidiomycetes- Amplification to the identification of mycorrhizae and ruts. *Mol Ecol* **2**: 113–118.

**Matheny PB, Liu YJ, Ammirati JF, Hall BD. 2002**. Using RPB1 sequences to improve phylogenetic inference among mushrooms (*Inocybe*, *Agaricales*). *Am J Bot* **89**: 688–698.

**Rehner SA, Samuels GJ. 1994**. Taxonomy and phylogeny of *Gliocladium* analysed from nuclear large subunit ribosomal DNA sequences. *Mycol Res* **98**: 625–634.

**Stiller JW, Hall BD. 1997**. The origin of red algae: implications for plastid evolution. *PNAS* **94**: 4520–4525.

**Vilgalys R, Hester M. 1990**. Rapid genetic identification and mapping of enzymatically amplified ribosomal DNA from several *Cryptococcus* species. *J Bacteriol* **172**: 4238–4246.

**White TJ, Bruns TD, Lee SB, Taylor JW**. **1990**. Amplification and direct sequencing of fungal ribosomal genes for phylogenies In: Innis MA, Gelfand DH, Sninsky JJ, White TJ, eds. *PCR Protocols: a guide of methods and applications*. New York: Academic Press, 315–322.

**Zoller S, Scheidegger C, Sperisen C. 1999.** PCR primers for the amplification of mitochondrial small subunit ribosomal DNA of lichen-forming ascomycetes. *Lichenologist* **31**: 511–516.

Supplementary Table 2. Best partitioning scheme and associated substitution models used in the different phylogenetic analyses as estimated with PartitionFinder.

| **Phylogenetic analysis** | **Best partitioning scheme** | **Nucleotide substitution models** |
| --- | --- | --- |
| *Stellarangia* spp. | (ITS1-2, *RPB1*_intron)  (58S)  (LSU, *RPB1*_exon_p1)  (*RPB1*_exon_p2, mtSSU)  (*RPB1*_exon_p3) | K80+I+G (ITS1-2, *RPB1*_intron)  K80 (5.8S)  K80+I+G (LSU, *RPB1*_exon_p1)  HKY+I (*RPB1*_exon_p2, mtSSU)  K80 (*RPB1*_exon_p3) |
| *Buellia* spp. | (ITS1-2)  (5.8S) | GTR+G (ITS1-2)  K80+I+G (5.8S) |

Supplementary Table 3. The BLAST results based on ITS data for the different lichen specimens of the genera *Stellarangia* (code S), *Xanthoparmelia* (X) and *Buellia* (B). The accession numbers of the closest matches in the GenBank database are provided, as well as the cover and identity percentages. An indication of the habitat (epilithic, E; hypolithic, H) of each specimens is given in the last two columns.

|  | Closest relative | Accesion  number | Cover  (%) | Ident.  (%) | Habitat  E H | |  |
| --- | --- | --- | --- | --- | --- | --- | --- |
|  | ITS |  |  |  |  | |  |
| S1 (AL8, AL38) | *Stellarangia elegantissima* | KT291454 | 100 | 96.6 | x | X | |
|  | *Stellarangia testudinea* | KC179312 | 86 | 99.8 |  |  | |
| S2 (AL49, AL59, AL36) | *Stellarangia elegantissima* | KT291454 | 100 | 96.7 |  | X | |
|  | *Stellarangia testudinea* | KC179312 | 86 | 100 |  |  | |
| S3 (AL17, AL30, AL58, AL47, AL53, AL32) | [*Stellarangia elegantissima*](https://blast.ncbi.nlm.nih.gov/Blast.cgi#alnHdr_914721953) | KT291454 | 100 | 96.3 | x | X | |
| S4 (AL23, AL25) | *Stellarangia elegantissima* | KT291454 | 100 | 97.6 | x |  | |
|  | *Stellarangia namibiensis* | KC179311 | 86 | 99.2 |  |  | |
| X1 | *Xanthoparmelia taractica* | MN103185 | 100 | 91.7 | x |  | |
| B1 (AL21) | *Buellia almeriensis* | MKF062519 | 84 | 83.85 |  |  | |
|  | *Buellia badia* | MK812426 | 64 | 91.3 | x |  | |
| B2 (Al62) | *Buellia russa* | DQ534454 | 100 | 86 |  | X | |
| B3 (AL35) | *Buellia frigida* | AY667583 | 99 | 88.1 |  | X | |

Supplementary Table 4. Species in family *Teloschistaceae* used in phylogenetic reconstructions of the genus *Stellarangia* and their GenBank accession numbers. Highlighted codes are those corresponding with sequences obtained in the present study.

|  | GenBank Accession No. | | | |
| --- | --- | --- | --- | --- |
| Species | ITS | LSU | mtSSU | *RPB1* |
| *Haloplaca* sp. | KC179295 | KC179203 | KC179537 | na |
| *Follmannia orthoclada* | KC179291 | KC179191 | na | na |
| *Leproplaca xantholyta* | KC179451 | KC179208 | KC179542 | na |
| *Sirenophila eos* | KC179300 | KC179246 | KC179585 | KT291581 |
| *S. gallowayii* | KC179301 | KC179247 | KC179586 | na |
| *S. jackelixii* | KC179303 | KC179248 | KC179587 | na |
| *Scutaria andina* | KC179298 | KC179242 | KC179581 | na |
| *Solitaria chrysophthalma* | KC179408 | KC179251 | KC179590 | KT291577 |
| *Stellarangia elegantissima* | KC179310 | KC179254 | KC179593 | KT291580 |
| *S. elegantissima* Cele75 | KT291454 | KT291541 | KT291488 | na |
| *S. namibensis* | KC179311 | Na | na | na |
| *S. namibensis* E156 | **MZ367686** | **MZ391147** | na | **MZ367573** |
| *S. namibensis* AL23 | **MZ367687** | **MZ391148** | na | **MZ367574** |
| *S. namibensis* AL25 | **MZ367688** | Na | **MZ363730** | na |
| *S. namibensis* OTU89 | **MZ367689** | Na | na | na |
| *Stellarangia* sp. AL17 | **MZ367690** | **MZ391149** | na | **MZ367575** |
| *Stellarangia* sp. AL30 | **MZ367691** | **MZ391150** | na | **MZ367576** |
| *Stellarangia* sp. AL32 | **MZ367692** | Na | na | na |
| *Stellarangia* sp. AL47 | **MZ367693** | Na | na | na |
| *Stellarangia* sp. AL53 | **MZ367694** | **MZ391151** | na | na |
| *Stellarangia* sp. AL58 | **MZ367695** | Na | na | na |
| *Stellarangia* sp. OTU214 | **MZ367696** | Na | na | na |
| *Stellarangia* sp. OTU119508 | **MZ367697** | Na | na | na |
| *S. testudinea* | KC179312 | Na | na | na |
| *S. testudinea* AL8 | **MZ367698** | **MZ391152** | na | **MZ367577** |
| *S. testudinea* AL36 | **MZ367699** | Na | na | na |
| *S. testudinea* AL38 | **MZ367700** | Na | na | na |
| *S. testudinea* AL49 | **MZ367701** | **MZ391153** | na | **MZ367578** |
| *S. testudinea* AL59 | **MZ367702** | Na | na | na |
| *S. testudinea* OTU69 | **MZ367703** | Na | na | na |
| *S. testudinea* OTU75002 | **MZ367704** | Na | na | na |
| *Teloschistopsis bonae-spei* | KC179322 | KC179257 | KC179596 | na |
| *T. eudoxa* | KC179324 | KC179258 | KC179597 | na |

Supplementary Table 5. Species in *Buellia* used in phylogenetic reconstructions and their GenBank accession numbers. Highlighted codes are those corresponding with sequences obtained in the present study.

| Species | ITS GenBank Accession No. |
| --- | --- |
| *Anaptychia ciliaris* | AY143391 |
| *Buellia* sp. | JX036043 |
| *Buellia* sp. AL21 | **MZ373309** |
| *Buellia* sp. AL27 | **MZ373310** |
| *Buellia* sp. AL28 | **MZ373311** |
| *Buellia* sp. AL29 | **MZ373312** |
| *Buellia* sp. AL35 | **MZ373313** |
| *Buellia* sp. AL62 | **MZ373314** |
| *Buellia* sp. AL67 | **MZ373315** |
| *Buellia* sp. AL68 | **MZ373316** |
| *Buellia* sp. AL71 | **MZ373317** |
| *Buellia almeriensis* | MF062519 |
| *B. aethalea* | AY143410 |
| *B. anisomera* | DQ534453 |
| *B. asterella* | AF250785 |
| *B. badia* | MK812426 |
| *B. capitis-regum* | AF540497 |
| *B. chujana* (Type) | NR_138413 |
| *B. dijiana* | AF250788 |
| *B. disciformis* | AY143392 |
| *B. elegans* | KX512901 |
| *B. erubescens* | KX512902 |
| *B. frigida* | KX512903 |
| *B. geophila* | MN615682 |
| *B. georgei* | AF250787 |
| *B. griseovirens* | AF540500 |
| *B. insignis* | MN615681 |
| *B. mamillana* | MN615693 |
| *B. numerosa* | LC153799 |
| *B. ocellata* | AF540502 |
| *B. papillata* | AF250790 |
| *B. polita* | MN615679 |
| *B. punctata* | KX512899 |
| *B. russa* | DQ534454 |
| *B. schaereri* | AF250791 |
| *B. subdisciformis* | AF352323 |
| *B. submuriformis* | AF540504 |
| *B. subnumerosa* | LC153800 |
| *B. subsororioides* | KM044008 |
| *B. taishanensis* | MG250190 |
| *B. tesserata* | KX512904 |
| *B. triseptata* | AF540506 |
| *B. zoharyi* | AJ421418 |
